# The Role of the Microbiota in Regeneration-Associated Processes

**DOI:** 10.3389/fcell.2021.768783

**Published:** 2022-01-26

**Authors:** Lymarie M. Díaz-Díaz, Andrea Rodríguez-Villafañe, José E. García-Arrarás

**Affiliations:** Department of Biology, University of Puerto Rico, Río Piedras Campus, San Juan, Puerto Rico

**Keywords:** regeneration, echinoderm, development, symbiosis, microbiota, microbiome, sea cucumber

## Abstract

The microbiota, the set of microorganisms associated with a particular environment or host, has acquired a prominent role in the study of many physiological and developmental processes. Among these, is the relationship between the microbiota and regenerative processes in various organisms. Here we introduce the concept of the microbiota and its involvement in regeneration-related cellular events. We then review the role of the microbiota in regenerative models that extend from the repair of tissue layers to the regeneration of complete organs or animals. We highlight the role of the microbiota in the digestive tract, since it accounts for a significant percentage of an animal microbiota, and at the same time provides an outstanding system to study microbiota effects on regeneration. Lastly, while this review serves to highlight echinoderms, primarily holothuroids, as models for regeneration studies, it also provides multiple examples of microbiota-related interactions in other processes in different organisms.

## Introduction

Microorganisms evolved billions of years before animals (reviewed in Knoll, 2003). It is now widely accepted that these microorganisms shaped the environment in which animals evolved (Szathmáry and Smith, 1995; Narbonne, 2005; Knoll, 2011). As a result, animals have conserved close associations with microorganisms, making the microbes an integral part of the animal’s environment. In recent years our understanding of the relationship between animals and microorganisms has advanced greatly, thanks in part to new technologies, such as sequencing technologies and mass spectrometers. These advances have brought with them new or redefined terms to describe the participants and/or relationships (Turnbaugh et al., 2007). Terms such as “microbiota” to describe the microbial taxa composition that are found within a certain environment, and “microbiome” to describe the collective genome of such symbionts (Turnbaugh et al., 2007) are now commonly used, and will be part of the terminology used in this review. Naturally, the impacts of the microorganisms have been, for many centuries, associated with disease. However, during the last decades, many studies have shown hitherto unrecognized roles, such as, protecting against pathogens (Iacob et al., 2019), modulating host metabolism, digestion, and nutrition (Kellow et al., 2013; Vijay-Kumar et al., 2010; Turnbaugh et al., 2006; O’Hara and Shanahan, 2006; Turnbaugh et al., 2009; Long et al., 2017), and immune system response (Neish, 2009; Round and Mazmanian, 2009; Bäckhed and Crawford, 2010; Fraune and Bosch, 2010). For example, it is now well established that an altered gut microbial ecosystem impairs gut homeostasis and health. Accordingly, an imbalance (dysbiosis) in the gut microbial community has been associated to diseases such as obesity (Ley et al., 2006; Turnbaugh et al., 2008), malnutrition (Kau et al., 2011), atherosclerosis (Karlsson et al., 2012), and diabetes type 2 (Qin et al., 2012) demonstrating the importance of the gut microbiota composition.

Genomic and molecular approaches, and the characterization of the microbiota role have allowed for new discoveries that extend beyond host health/disease issues (Weinstock, 2012). Recently, the microbiota has been associated with host development, including processes that were thought to be dependent on the host’s genetic program, such as morphogenesis and organ development (Sommer and Bäckhed, 2013). Moreover, it has been proposed that the microbiota might play roles in behavior, reproduction, and even in degenerative diseases, among others (Wang et al., 2017).

The present review focuses on the relationship between the microbiota and the process of regeneration. This is a relatively new area of research that explores how the associated microbial taxa within a particular host might modulate the regeneration of a particular tissue, organ or even the whole-body of the host species. We include a summary of models that have been used to study the role of the gut microbiota during intestinal regeneration and associated processes (Table 1). Therefore, for the writing of this review, we screened for articles relevant to our topics in the search engine PubMed (pubmed.ncbi.nlm.nih.gov) using keywords such as “microbiota”, “microbiome” and “regeneration”, among others, and included information considered pertinent. Specifically, we have highlighted research done in animals that belong to the phylum Echinodermata, a phylum known for extraordinary regeneration abilities such as partial or total re-growth of different appendages or internal organs (García-Arrarás and Dolmatov, 2010). In particular, many of them are able to regenerate their digestive tract, thus providing the venue to study the effect of the microbiota in one of the organs best known for microbiota-host associations. This review serves to present a group of echinoderms, the holothurians or sea cucumbers, as excellent models to study microbiome-host associations and their impact on regenerative processes.

**TABLE 1:** Model systems used to decipher the associations between the microbiota and the intestinal regeneration in biomedical research.

Model system	Hallmarks of the model	Microbial association	Limitations of the model	References
Planarian	Display whole body regeneration	Pro- and anti- regenerative properties of *Pseudomonas* and *Aquitalea* sp in whole body regeneration. Apoptosis regulation	Intestinal regeneration cannot be separated from whole body regeneration	Arnold et al. (2016), Lee et al. (2018)
Fruit flies (*Drosophila melanogaster*)	Have the basic structure of the digestive system with simpler microbial communities. Ease of studying roles of the microbiome in the modulation of host signaling pathways and physiology	Microbial community modulates stress response and promotes stem cell proliferation and epithelial regeneration. Specifically, *Erwinia carotovora* was shown to help intestinal epithelial repair	Invertebrate/Protostome. Limited to intestinal epithelial homeostasis and renewal. It was suggested that *Drosophila* gut structure allows oxygen to circulate across the tract, which differs from vertebrates	Shin et al. (2011), Buchon et al. (2009), Chandler et al. (2011), Charroux and Royet (2012)
Zebrafish (*Danio rerio*)	Vertebrate model to study roles of the microbiome in the modulation of host signaling pathways and physiology	*Aeromonas veronii* and *Helicobacter pylori* facilitate epithelial cell proliferation. Microbiota was also shown to promote intestinal epithelial cell fate determination	Only the regeneration of the intestinal luminal epithelium has been studied	Bates et al. (2007), Cheesman et al. (2011), Neal et al. (2013), Rawls et al. (2004)
Rodents	Mammal models to study the gut microbiota in the intestine	The microbial community contributes to the modulation of intestinal epithelial cell proliferation, differentiation, and migration. Microbiota promotes tissue regeneration through induction of the immune system	Only the regeneration of the intestinal luminal epithelium has been studied	Hou et al. (2017), Thomas (2016), Sommer and Bäckhed (2013), Pellegatta et al. (2016), Abrams et al. (1963), Uribe et al. (1994)
Isolated cells/cell lines (mammal models)	Easy handling and maintenance	The microbial community contributes to the modulation of intestinal epithelial cell proliferation, differentiation, and migration. *Akkermansia muciniphila* and *Lactobacillus rhamnosus* are associated with epithelial wound healing	2D model of isolated cells, lacks the composition and integrity of the intestine	Alam and Neish (2018), Hooper et al. (2001), Pull et al. (2005), Rakoff-Nahoum et al. (2004), Lam et al. (2007), Alam et al. (2016), Swanson et al. (2011)
Organoids (mammal models)	Non-invasive methods to study the microbial community in mammals. Share the cellular and structural composition, as well as the self-renewal dynamics, of the intestinal epithelium	*Lactobacillus reuteri* protects the morphology of intestinal organoids and normal proliferation. Proliferation and differentiation occurred through a TLR4-dependent pathway triggered by bacterial-derived LPS	Reduced view of the digestive system, limited to cells from intestinal lineage	Lancaster and Knoblich (2014), Hou et al. (2017), Hou et al. (2018), Naito et al. (2017)
Sea cucumber *Holothuria glaberrima*	Deuterostome model. Has the basic structure of the digestive system with simpler microbial communities. Can regenerate the small and large intestine upon evisceration. The cellular events that control the regeneration have been well characterized	Antibiotics delayed the intestinal regeneration. Gram-positive bacteria (Firmicutes and Actinobacteria) may have a crucial role in the progression of their intestinal regeneration	Marine invertebrate ecosystem. Few studies characterizing the microbiota and their possible roles during the regeneration process	García-Arrarás et al. (1998), Quiñones et al. (2002), Mashanov et al. (2005), Candelaria et al. (2006), García-Arrarás et al. (2011), Mashanov and García-Arrarás (2011), García-Arrarás et al. (2018), Quispe-Parra et al. (2021), Pagán-Jiménez et al. 2019), Díaz-Díaz et al. (2021)

**FIGURE 1:**
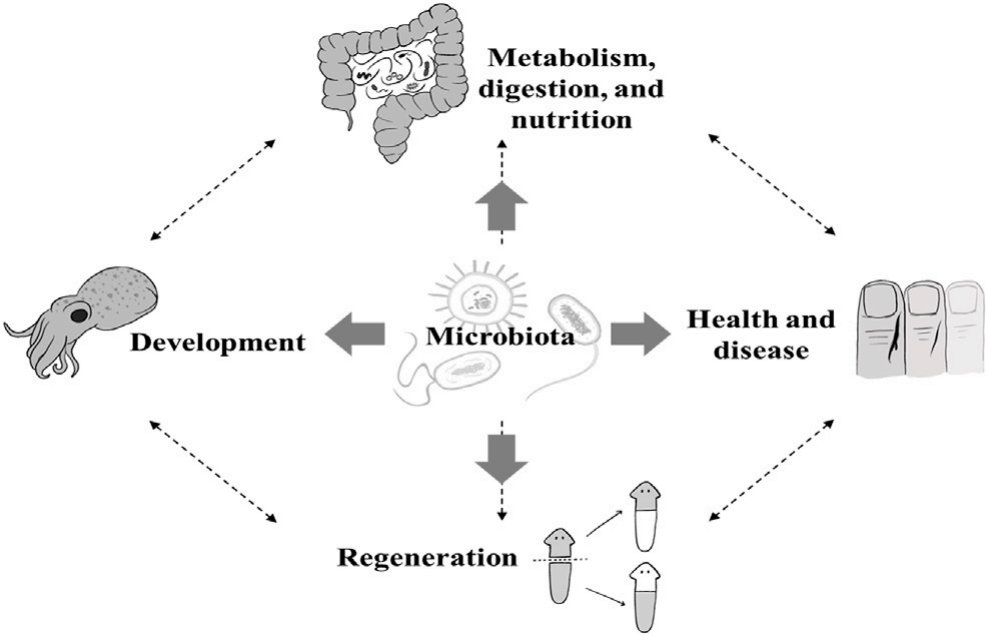
*The influence of microbiota on host physiology.* This figure outlines the aim of this review where we describe the role of the microbial composition associated with an animal host. In this review we focus on the regeneration process. However, we incorporated studies that link the microbiota to the metabolism, digestion and nutrition, health, and development of animal hosts to point out the interconnection between all these processes (dashed arrows).

Prior to delving into microbiome-regeneration studies, we begin by reviewing some findings from three regeneration-related fields where microbiome associations are important to the host. These are the association of the microbiota with: 1) the host metabolic/digestive processes, 2) embryonic developmental processes, and 3) wound healing (Figure 1). These three processes play important roles in regeneration and two of them (wound healing and embryonic development), share key mechanisms with regeneration, thus, the particular interest in singling them out.

### Microbiota is Essential for Host Metabolism, Digestion, and Nutrition

From the roles ascribed to the microbiota, probably the best understood is their importance on host metabolism, which impacts their digestion and nutrition, by the assimilation of the digested food for the host physiological process. Multiple studies have shown the involvement of the mammalian gut microbiota in metabolic processes and energy homeostasis of host animals (O’Hara and Shanahan, 2006; Turnbaugh et al., 2006; Turnbaugh et al., 2009; Vijay-Kumar et al., 2010; Long et al., 2017). The gut microbiome was found to be crucial in processing non-digestible substrates that are necessary for host health maintenance and thus physiology (Gill et al., 2006). For example, the fermentation of dietary fibers and endogenous intestinal mucus, ensured by the intestinal microbiota, allows the growth of microorganisms that produce short-chain fatty acids (SCFAs) and gases (Wong et al., 2006). Acetate, the most abundant SCFA, is used in cholesterol metabolism and lipogenesis in the peripheral tissues (Frost et al., 2014). Butyrate, another major SCFA, is the main energy source for human luminal cells in the colon (De Vadder et al., 2014), and is key for generating a hypoxia state in epithelial cells, oxygen balance, and prevention of gut microbiota dysbiosis (Byndloss et al., 2017). The butyrate producer *Faecalibacterium prausnitzii*, one of the most represented bacteria in the intestine of healthy human adults, has exhibited anti-inflammatory effects in a colitis-mouse model (Miquel et al., 2013)*.* Propionate, another dominant SCFA, regulates gluconeogenesis and satiety signaling through interaction with the gut fatty acid receptors in the liver (De Vadder et al., 2014). Another example of bacteria metabolites that can alter a host’s physiology is polyhydroxybutyrate (PHB), which is a polyhydroxylkanoate that comprises the primary product of carbon assimilation from glucose and starch. Microorganisms retain PHB and metabolize it when other common energy sources are not available, principally when carbon concentration is higher than nitrogen’s (Madison and Huisman, 1999; Jendrossek and Pfeiffer, 2014). Moreover, PHBs are used for host development both in fish and crustacean aquaculture (De Schryver et al., 2010; Nhan et al., 2010; Najdegerami et al., 2012).

The data shown above, focusing on a minor subset of gut bacterial products, clearly present the interdependence of the microbiota with its host highlighting how bacterial metabolites are not only essential for the host physiological processes but are also needed for the growth of other bacteria.

### Microbiota Role in Development: Focus on Immune System and Organ Formation

*Immune system development and activation*- The actions of some symbionts go well beyond localized functions and are crucial for the overall development of the host. This provides a useful background for our discussion of microbiota effects on regeneration, specifically because of the links between embryological development and regeneration. Multiple studies from different organisms have demonstrated that the cellular and molecular mechanisms used in regenerative processes are similar, and in many cases identical, to those that take place during development (Arvizu et al., 2006; Yokoyama et al., 2008; Petersen and Reddien, 2009; Tu and Johnson, 2011; Tischer et al., 2013; Bryant et al., 2017; Reddy et al., 2019).

Therefore, the role of the microbiota during an organism’s developmental history can lead to important insights on a possible role during regeneration processes in the same or closely related organisms. A classic example of the effect of microbiota during embryonic or postnatal development is the development of the immune system in vertebrates (Round and Mazmanian, 2009; Bäckhed and Crawford, 2010; Fraune and Bosch, 2010). Studies have revealed that mutualistic or commensal microbe colonization are pivotal for the development, maturation, and activation of the immune system. Developmental effects of the microbiota in vertebrate species have usually been studied using germ-free models. In some of the key studies, germ-free reared animals presented deficient development of the immune system, including underdeveloped lymphatic organs (Falk et al., 1998; Macpherson and Harris, 2004; Bouskra et al., 2008), and defects in T cell regulation and B cells antibody production (Round and Mazmanian, 2009). In addition to the direct effects of these symbionts through the production of antimicrobial substances, immune response in germ-free animals lacked a priori instruction, induced by commensals (Hansen et al., 2014). This was confirmed with the propensity to infections when microbes were reintroduced to germ-free animals.

The study of Toll-like receptor (TLR) signaling in host-microbiome models has shown the mechanism by which the microbiota interacts with immune system activation and maturation (Akira and Takeda, 2004). This pathway is highly conserved in metazoans (Khalturin et al., 2004; Iwanaga and Lee, 2005; Roach et al., 2005; Satake and Sekiguchi, 2012; Nie et al., 2018), increasing the number of possible models in which to examine the relationship between microbiota and host immunity. The Toll pathway is activated by the binding of various microbe-associated molecular patterns (MAMPs) to the Toll-like receptors (TLRs) (Janssens and Beyaert, 2003; Kawai and Akira, 2010; Narayanan and Park, 2015; Wang et al., 2020). In some invertebrates, the pathway is activated indirectly, when the cytokine-like endogenous molecule Spätzle detects the microorganisms and activates the Toll receptors (Kawai and Akira, 2010). The activation of Toll pathway provokes the secretion of toxic molecules, such as antimicrobial peptides and reactive oxygen species (ROS) (Tzou et al., 2000; Ha, EM, et al., 2005).

Studies in mice have identified possible mediators of the microbiota-host immune response. These studies revealed that mice harbor specific Firmicutes, *Candidatus arthromitus* (Snel et al., 1995), that influence the innate immune system maturation (Suzuki et al., 2004; Gaboriau-Routhiau et al., 2009; Ivanov, II, et al., 2009). This suggests that Fusobacteria and Firmicutes may be important in the regulation of immune system development, immune-inflammatory response, and gut homeostasis. However, these filamentous bacteria have only been found in some infants younger than 3 years old (Yin et al., 2013), and a similar role in immune maturation in humans remains to be discovered. Moreover, recent studies have evidenced that metabolite generation, including SCFAs and adenosine triphosphate, influences the host’s immunity (Atarashi et al., 2008; Furusawa et al., 2013).

*Organ morphogenesis-* That the microbiota is involved in the process of immune system development and maturation might be expected, since after all, one of the system’s main functions involves the direct interaction of immune cells with the environmental bacteria. Other findings that associate the microbiota with an organism’s development are somewhat more surprising. One such study is the symbiotic association between the marine bobtail squid *Euprymna scolopes* and bioluminescent *Vibrio fischeri.* This model has arguably played a pivotal role in advancing the field of host-microbe associations involved in developmental processes (Nyholm and McFall-Ngai, 2004). This model provides an interesting phenomenon where the host-microbiome interaction is crucial to the formation of an anatomical complex structure and at the same time is not associated with health/disease issues, as are most other cases involving the microbiota. In this system, during development, the squid forms a structure named “the light organ” which helps in the protection of the host from predators (Boettcher and Ruby, 1990; McFall-Ngai and Ruby, 1998; Jones and Nishiguchi, 2004). This organ is colonized by bacteria during the day, the photosynthetic bacterium camouflages the squid from predators at night, and then at dawn, the squid ejects the light organ bacteria into the ocean, a cycle that is repeated daily.

Researchers have described in detail the process of host colonization and bacterial interactions (Nyholm and McFall-Ngai, 2004). Newly hatched juveniles are born with fields of ciliated epithelia on the nascent squid rudimentary light organ (McFall-Ngai and Ruby EG, 1991). They acquire the bacteria from the ocean environment (Ruby and Lee, 1998). When the host is exposed to bacterial peptidoglycan, the epithelial cells produce mucus that promotes the aggregation of bacteria (Nyholm et al., 2000; Nyholm et al., 2002). The symbiont then moves in the mucus to the crypt spaces of the light organ and colonizes it. As a result, it triggers developmental changes of the squid light organ (Doino and McFall-Ngai, 1995). Some of these adaptations include constriction of the ducts that lead to the crypt space delimitation, suspension of mucus secretion, and a regression of the ciliated epithelium, which might prevent further colonization of environmental symbionts. Other changes include trafficking of hemocytes into the blood of the ciliated epithelium (Nyholm and McFall-Ngai, 2004) facilitating the retrogression of the ciliated epithelium (Koropatnick et al., 2007), and increasing the density of microvilli in crypt cells (Nyholm and McFall-Ngai, 2004) which increases the surface area of interaction between the bacteria and the crypt cells (Lamarcq and McFall-Ngai, 1998). In addition to morphological and mechanical adaptations, chemical changes also take place. For example, following colonization by *V. fischeri* during crypt metamorphosis, a decrease in nitric oxide (NO) production is observed (Davidson et al., 2004). All these events favor the *V. fischeri* selection and proliferation to ensure mature organ light formation and bioluminescence (Nyholm and McFall-Ngai, 2004).

Antibiotic induced *V. fischeri* clearance from the crypts produces some irreversible developmental changes, such as the permanent loss of the surface ciliated epithelium and the attenuation of NO in the ducts (Nyholm and McFall-Ngai, 2004). Mutant *V. fischeri* that are defective in producing luminescence because of a mutation in the luxA gene (Visick et al., 2000) or deletion of lux operon do not persist in the crypts (Bose et al., 2008). Apart from not producing the required luminescence, these mutants cause developmental effects on the host, which fail to appropriately induce swelling of the crypt epithelial cells, hemocyte trafficking, and apoptosis of cells of the epithelial fields (McFall-Ngai et al., 2012). The mutant bacteria also have an altered expression of lipopolysaccharide (LPS) lipid A and peptidoglycan (PGN) tracheal cytotoxin (TCT) monomer. This correlates with observed changes in squids exposed to mutant bacteria that have a different expression of their LPS-binding proteins and peptidoglycan-recognition proteins. Thus, *V. fischeri*’s luminescence is somehow dependent on the expression of MAMPs and host pattern-recognition receptors to induce the immune system to cause the developmental changes in *E. scolopes* (McFall-Ngai et al., 2012).

Another interesting aspect of this symbiosis is the fact that *V. fischeri* that colonize the light organ are not eliminated by the immune system of *E. scolopes* (McFall-Ngai et al., 2012). It is thought that the recognition of *V. fischeri* molecules play a pivotal role in the selection of bacterial species by the immune system and therefore, the morphogenesis of the light organ of the host (Koropatnick et al., 2004; Troll et al., 2010).

*Other developmental effects-* Microbiota effects on embryonic development have been studied in other invertebrates (these are usually chosen because they generally have simpler microbial communities). The *Drosophila-Acetobacter* system has been a convenient model for understanding the genetic and functional roles of the microbiome in the modulation of host signaling pathways and physiology. Extensive studies in *Drosophila* and its symbiont *Acetobacter pomorum* showed that this gut bacteria impacts not only the metabolism of its hosts, but the growth, body size gain, and stem cellular activity (Shin et al., 2011). An *A. pomorum* mutant library has been used to decipher their beneficial role on host’s developmental homeostasis. This has led to the finding that the periplasmic pyrroloquinoline quinone–dependent alcohol dehydrogenase (PQQ-ADH)–dependent oxidative respiratory chain of the *A. pomorum* interaction with the insulin/insulin-like growth factor (IGF)-1 signaling (IIS) of the host is necessary for the maintenance of the gut mutualism. However, the sole bacterial PQQ-ADH is insufficient to promote the *A. pomorum*–mediated effects on host physiology, suggesting that the host genetic program and gut bacteria regulate each other. Additional studies using multiple insect models have confirmed the role of the hindgut bacteria in various aspects of digestion and host development. These cases include digestive efficiency of soluble plant polysaccharides and growth rate in crickets (Kaufman and Klug, 1991), insect generation time, adult body weight gain, and methane production in cockroaches (Gijzen and Barugahare, 1992), cellulose breakdown and nitrogen fixation in beetles (Morales-Jiménez et al., 2009), and potential proteolytic activity in aphids (Wang and Zhang, 2015).

Many studies performed in germ-free mammals have shown that the intestinal microbiota influences the postnatal development of the gastrointestinal tract in these organisms. For example, in mice, successions in the microbiota composition during development were shown to lead to gastrointestinal maturation (Wagner et al., 2008; Reinhardt et al., 2009). The intestine of an adult mouse accommodates a sophisticated vascular network that originates from a system of vessels that form postnatally in small intestinal villi. The formation of this network occurs concurrent with the assembly of the microbiota. Comparative studies of the capillary networks of germ-free mice versus animals colonized (ex-germ-free) during or after gut development demonstrated abnormalities in the capillary network of adult germ-free mice (Stappenbeck et al., 2002). However, colonization either with conventionalized mice microbiota or with *Bacteroides thetaiotaomicron* restarted and completed the developmental program. Other studies, using germ-free transgenic mice lacking Paneth cells (which secrete antibacterial peptides that affect luminal microbial ecology) in the intestinal epithelium, showed that this angiogenesis was regulated by *B. thetaiotaomicron* colonization of the mucosal surface (Stappenbeck et al., 2002). In addition, the associated microbial community contributes to the modulation of intestinal epithelial cell proliferation, as evidenced by the scarcity of proliferating cells in the intestines of germ-free rodents (Abrams et al., 1963; Uribe et al., 1994) and zebrafish (Rawls et al., 2004).

Further information on the role of the microbiota on vertebrate developmental processes has been obtained using germ-free and gnotobiotic zebrafish (Milligan-Myhre et al., 2011). Both zebrafish and murine germ-free models presented significant differences in the intestinal morphology in comparison with conventional controls, including reduced cell division, decreased number of goblet cells and intestinal associated immune cells, and perturbed expression of genes involved in metabolism and innate immunity (Savage et al., 1981; Kandori et al., 1996; Cebra et al., 1998; Hooper et al., 2001; Rawls et al., 2004; Bates et al., 2007; Bouskra et al., 2008; Cheesman et al., 2011; Kanther et al., 2011).

### A New Role for Microbiota as Regulator of Regenerative Processes

The role of the microbiota has been studied, quite extensively, in processes associated with wound healing. These processes are usually the initial steps in more complex regenerative events, and will be briefly reviewed here, prior to discussing the role of the microbiota in overall regeneration of tissues and organs.

*Wound healing following injury* - The first response after a trauma or injury to an organism is the wound healing cascade which ensures the repair of the wound and avoids the colonization or translocation of pathogens. This takes place prior to the reorganization of the injured tissue (Guo and Dipietro, 2010) and might involve the microbiota (Thomas, 2016; Maheswary et al., 2021) as shown by studies of human skin microbiota during wound healing processes. Many of the findings on the role of microbiota in wound healing were facilitated by studies of chronic wounds, such as diabetic foot ulcers and non-healing surgical wounds, which represent major healthcare problems. These studies provided valuable data on how the microbiota can shape the process of wound healing and perhaps other processes related to regeneration.

Chronic wounds are caused by a disruption of the cutaneous wound healing process, preventing the restoration of the skin barrier. The main bacterial phyla identified in acute and chronic wounds are also found in healthy skin, however wounds are characterized by skin dysbiosis where their relative abundance differs significantly by wound type (Ammons et al., 2015; Loesche et al., 2017). *Pseudomonas* and *Staphylococcus* dominate in all types of chronic wounds (Dowd et al., 2008; James et al., 2008; Gardner et al., 2013; Wolcott et al., 2016; Gardiner et al., 2017), and usually are present in acute wounds created by blunt or penetrating trauma (Hannigan et al., 2014; Bartow-McKenney et al., 2018), burns (Hannigan et al., 2014; Liu et al., 2018), or atopic dermatitis (Seite et al., 2014). However, higher levels of anaerobic bacteria are present in chronic wounds and are commonly associated with worse prognosis (Loesche et al., 2017).

Moreover, pathogenic microorganisms are suspected of playing a substantial role in delayed wound healing. Hence, perturbations of microbial communities that are not promoting cutaneous wound healing may be beneficial. As shown by Loesche et al. the use of antibiotics to destabilize pathogenic wound microbiomes, resulted in faster wound healing (Loesche et al., 2017). In other studies, when probiotic bacteria were applied to a rodent wound, the bacterial load was decreased and tissue repair was promoted (Rodrigues et al., 2005; Valdez et al., 2005; Huseini et al., 2012). Similarly, wounded dermal tissues of mice showed improved proliferation of epidermal cells, vascularization, and re-epithelialization after inoculation with *Pseudomonas aeruginosa* strain PAO1 (Kanno et al., 2011). Also, in humans, topical application of probiotics exerted positive wound healing properties for chronic venous ulcers infected with *Staphylococcus aureus* and *Pseudomonas aeruginosa* (Peral et al., 2009). Consequently, microbial communities may be useful for the diagnosis of wound healing progresses (by predicting those wounds that will experience infectious complications). Hence, studies in skin microbiota provide an example of interactions between host and microbiomes with biomedical relevance to health issues.

In addition, the gut microbiota has been implicated in intestinal epithelial repair. This is highlighted by recent studies on intestinal wounds where gut microbiota enhanced epithelial wound repair (Alam and Neish, 2018). Specifically, intestinal commensal bacteria have been found to regulate the proliferation, migration, and survival of host epithelial cells, as well as promote barrier function and resolution of epithelial wounds (Hooper et al., 2001; Rakoff-Nahoum et al., 2004; Pull et al., 2005; Lam et al., 2007). One of these commensals is *Akkermansia muciniphila*, which is enriched in healing mucosal wounds and dominates the wound-mucosa-associated microbiota (Alam et al., 2016). When mice are treated with exogenous *A. muciniphila* to treat colonic mucosal wounds enhanced mucosal closure occurs. The bacterial treatment stimulates the mice intestinal cellular proliferation and enterocyte migration from the crypt apparently through the generation of ROS when the bacteria colonize the wounded area. The possibility that ROS might be the mediator in this phenomenon is strengthened by experiments with another gut commensal, *Lactobacillus rhamnosus.* This bacterium has also been associated with intestinal epithelium repair by experiments showing that the sole contact of intestinal epithelial cells (IECs) with *L. rhamnosus* strain GG (LGG) induces ROS accumulation, consequently stimulating cellular proliferation and migration (Swanson et al., 2011).

**FIGURE 2:**
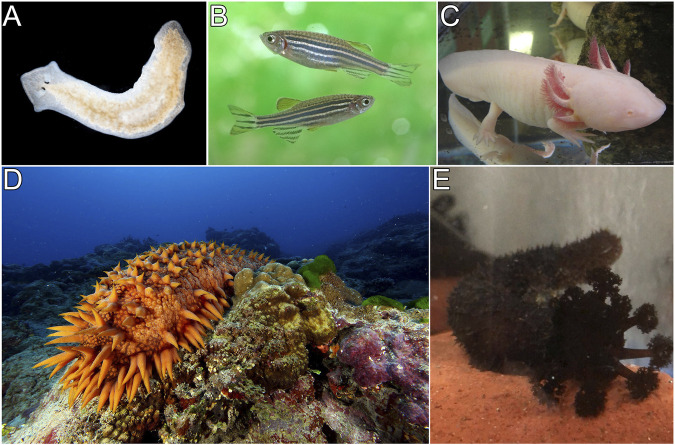
*Models of regeneration.* This figure portrays organisms that are used as regeneration models: planaria **(A)**, zebrafish **(B)**, axolotl **(C)**, and two holothurian species, *Apostichopus japonicus* **(D)** and *Holothuria glaberrima* **(E)**.

Metazoans have different regeneration capabilities. Since mammals are not well know for their regenerative potential, the roles of microbiota in the regeneration of tissues or organs have been focused on particular model organisms. Various species, well known for their regenerative responses, such as planarians, salamanders, and zebrafish have been used to study whether the microbiome can regulate the regeneration potential of their hosts or are directly involved in the regeneration process (Figure 2). Some of these roles will be discussed below.

*Whole body regeneration in planarians*- Two studies in the planaria *Schmidtea mediterranea* have shown that bacteria can influence whole body regeneration. In the first study, the microbiome of healthy planarians was characterized, revealing a high Bacteroidetes to Proteobacteria ratio (Arnold et al., 2016). Animal manipulations such as tissue amputations and changes in culturing conditions (which elicits a relative increase of Proteobacteria) and cultures with a strain of *Pseudomonas*, produced ectopic lesions and progressive tissue degeneration. Furthermore, infection with the *Pseudomonas* strain enhanced apoptosis, in contrast to what occurs in the absence of infection where regeneration represses apoptosis. To explain this phenomenon, Arnold et al. suggested that activation of an innate immunity signaling (TAK1/MKK/p38) pathway had an opposite role in host immunity versus normal regeneration. In a second study, a different group studied the impact of bacterial metabolites on the regeneration of planarians (Lee et al., 2018). They described the microbial community of *Dugesia japonica*, a close relative to *S. mediterranea,* and inoculated tail and head-amputated antibiotic-treated organisms with representative bacteria species. Lee and colleagues found that regeneration was compromised in animals inoculated with an indole producing bacteria, *Aquitalea* sp., and tail and head formation was delayed. To test whether the production of indole (which is formed from tryptophan by bacterial enzymatic action) was the causative agent, amputated trunks were incubated with *Aquitalea* sp. in tryptophan supplemented media. Animals exposed to both tryptophan and indole producing bacteria presented a delayed regeneration in comparison to controls. These experiments demonstrated a direct effect of an indole-producing bacteria on the regenerative properties of planarians.

*Limb regeneration in salamanders*- A possible association between bacteria and regeneration has also been observed in one of the best studied vertebrate regeneration models, the Mexican axolotl *Ambystoma mexicanum* (Demircan et al., 2019). This amphibian is capable of regenerating internal organs such as heart, brain, and lungs and external organs such as limbs, gills, and tail (Vieira et al., 2020). In Demircan and colleagues work, a 16S rRNA amplicon dataset was obtained from limbs at different days post amputation (dpa) and correlated with axolotl limb regeneration stages; the stages (0-, 1-, 4-, 7-, 30-, and 60- dpa) (Demircan et al., 2019). Although the study was purely correlative, it showed changes in the microbiota during regeneration, suggesting that certain bacterial groups might be associated with the regenerating tissues. At the phylum level, the bacterial communities in normal animals were dominated by Bacteroidetes, Firmicutes, Proteobacteria, Actinobacteria, and Verrucomicrobia. In regenerating limbs, a temporal shift in bacterial composition was observed, which included differential phylum abundances at certain limb regeneration stages. Post-amputated groups had different microbial communities compared to aquarium control groups, since there was a shift from Firmicutes-enriched (controls) to Proteobacteria-enriched (regenerating) relative abundance. The significant differences observed between the water and the regenerating limb microbiotas suggested selective colonization of axolotl limb tissues and that substantial restructuring of bacterial communities occur in regenerating tissues. Moreover, a comparison of the microbial community demonstrated less variation in the relative abundance of bacterial communities between samples at the same stage of regeneration, and higher variation between groups at different stages. Also, they found differences between limb microbial communities among the regeneration phases: the 0- and 1- dpa samples, 4- and 7- dpa samples, and 30- and 60- dpa samples all differed between them in the measures of beta-diversity. That different bacterial communities were found at specific limb regeneration stages, such as wound healing, dedifferentiation, and re-development, could indicate that specific bacterial groups have specific roles in these processes.

**FIGURE 3:**
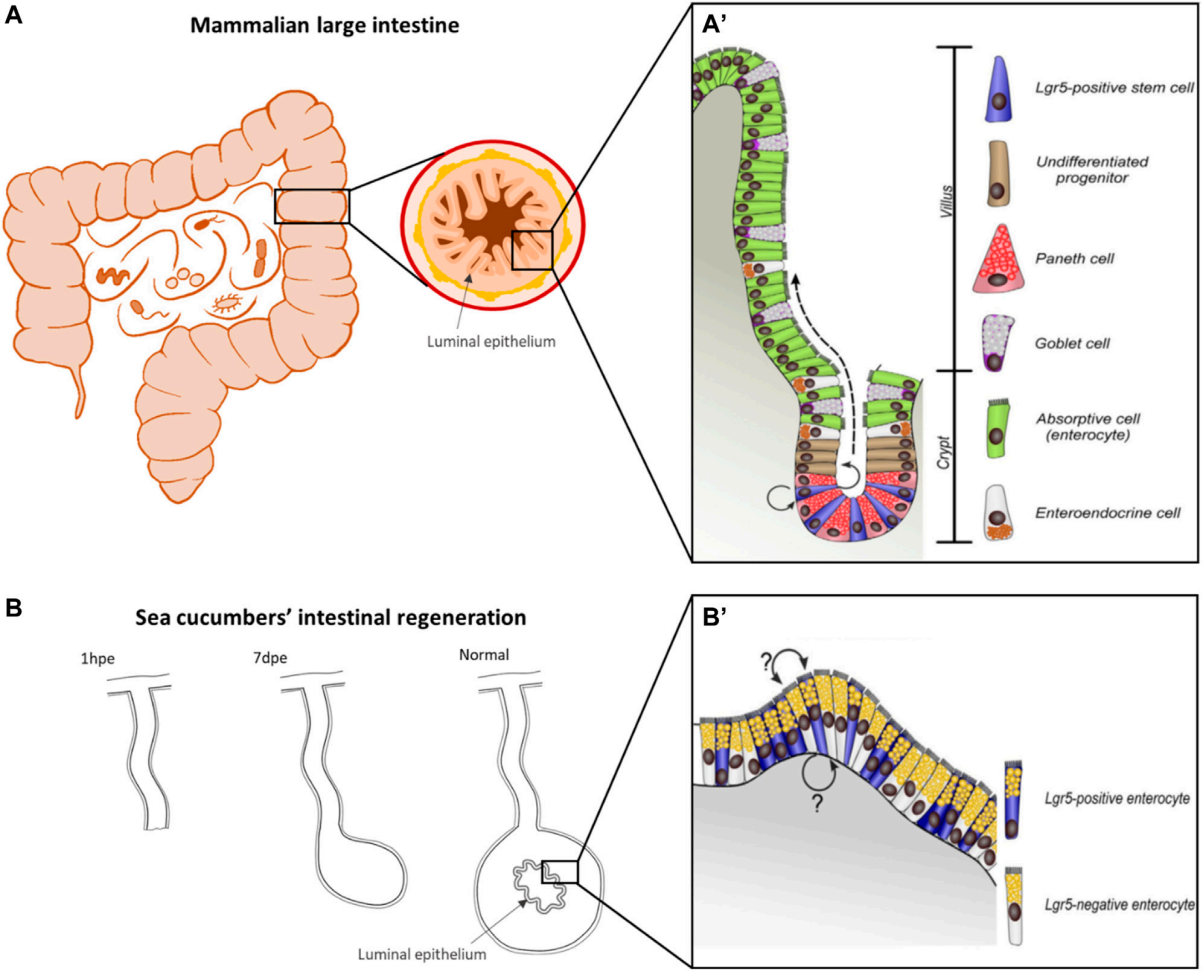
*Comparison of mammalian and holothurian intestinal epithelium anatomy and renewal.* Representative organization of the luminal epithelium of mammal intestine **(A)** and the mucosal epithelium in the digestive tube of sea cucumbers **(B)**, highlighting the difference in cell renewal mechanisms. **(A)** In mammals, Lgr5-positive intestinal stem cells are localized at the bottom of the crypt, which self-renew and produce dividing transit-amplifying progenitors (arrows), which gradually migrate apically and form the villus (dashed arrow), where are localized the specialized cells. Paneth cells (which appear to be unique in mammals) are the only differentiated cell type that remains in the stem cell niche. **(B)** In the digestive epithelium of echinoderms, the spatial organization of mammals is not present, instead Lgr5-positive cells are interspersed among Lgr5-negative or differentiated cells, but the lineage of these cells is not well understood. **(A′,B′)** were retrieved from Mashanov et al., 2014 and modified by LD-D, **(A,B)** are drawings by the authors of this article (AR-V and LD-D, respectively) for the purposes of this comparison.

*Tissue layer (luminal epithelium regeneration) in vertebrates-* Many investigators have studied the regeneration of the luminal epithelial layer (Figure 3A) of the vertebrate digestive tract (see Santos et al., 2018 for review). This tissue layer is continuously being formed as the cells undergo damage by the exposure to the digestive lumen content and the digestive process itself (Barker et al., 2007; Sailaja et al., 2016; Santos et al., 2018). In addition to the ongoing epithelial turnover to achieve gut homeostasis, this tissue can undergo regeneration if injured by exposure to factors such as toxins, radiation or others (Metcalfe et al., 2014; Beumer and Clevers, 2016). Homeostatic maintenance of the luminal epithelium is well understood and has been well described particularly in the mammalian intestine (Barker et al., 2007). The renovation of the layer is dependent on the intestinal stem cells (ISC) and their associated environment (ISC niche). These cells are found within the luminal crypts and give rise to the different cell types in the epithelium. The stem cells divide within the crypts and their progeny continue this division as they transit to the intestinal villi where they differentiate into the intestinal luminal epithelial phenotypes. As cells reach the tip of the intestinal villi, they are shed into the lumen, maintaining a continuous migration of cells from the crypts to the villi.

As response to injury, the ISC niche adapts to ensure epithelial regeneration beyond the homeostatic state (Beumer and Clevers, 2016). The epithelial restitution is achieved either by proliferation of active ISCs (Lgr5^+^ ISCs) or by mature cells dedifferentiated to ISC. This regeneration of mucosal epithelia has been found to be modulated by the microbiota (Thomas, 2016; Hou et al., 2017). Also, the microbiota has been suggested to promote gut healing regeneration through induction of immune responses (Sommer and Bäckhed, 2013; Pellegatta et al., 2016; Thomas, 2016; Hou et al., 2017).

Various experiments demonstrate a similar role of microbiota on luminal epithelium regeneration in zebrafish. For one, in the developing zebrafish intestine, epithelial cell proliferation was shown to be facilitated by their symbiont bacteria, *Aeromonas veronii* (Cheesman et al., 2011). In other studies, the virulence factor CagA from *Helicobacter pylori* also promoted intestinal cell proliferation through Wnt pathway signaling (Neal et al., 2013). Lastly, microbiota was also shown to promote intestinal epithelial cell fate determination via the Notch-MyD88 signaling (Bates et al., 2007; Cheesman et al., 2011).

Additional model systems, mainly *in vitro* models comprising cell cultures, tissue explants, and organoids, have been developed to decipher the microbiota’s influence on the homeostasis and regeneration of mammalian intestines. Among these, organoids have been used to understand the effects that the commensal microbiota, or a particular microorganism, might have on intestinal epithelium homeostasis (Peck et al., 2017; Blutt et al., 2019). Organoids are three-dimensional tissue structures obtained from stem cells in culture, that are differentiated into multiple organ-specific cell types. Thus, cells in these structures acquire some of the organ or tissue organization and functions (Lancaster and Knoblich, 2014). Small intestinal organoids share the cell and structural composition of the small intestinal epithelium, as well as the self-renewal dynamics. (Sato et al., 2009; Sato et al., 2011). Using organoids, it was shown that live *Lactobacillus reuteri* protected the morphology of intestinal organoids and normal proliferation (Hou et al., 2017; Hou et al., 2018). The protection of the intestinal barrier and activation of intestinal epithelial proliferation seemed to control intestinal inflammation.

A possible mechanism for the bacterial effect was described in a recent work showing that the ISC expresses nucleotide binding oligomerization domain-containing protein 2 (NOD2). This protein interacts with a peptidoglycan motif expressed on most bacterial organisms, suggesting a putative pathway for communication between the microbiome and the ISC niche (Nigro et al., 2014). Treatment of organoids with ligands for NOD2 resulted in an increase in their number and size, indicating that these ligands induce epithelial proliferation. Additional support that bacterial species have a role in the ISC niche comes from studies in mice, where a crypt-specific microbiome has been associated with homeostatic proliferation. This finding led to organoid studies showing that modulation of the colonic epithelial balance between proliferation and differentiation occurred through a TLR4-dependent pathway triggered by bacterial-derived LPS (Naito et al., 2017). Other work showed that colonic crypts from mice devoid of microbiota lose their regenerative capacity, as assessed by the ability to form organoids (Zaborin et al., 2017). There, the regenerative capacity was recovered by fecal microbiota transplantation that restored the crypt microbial communities. Furthermore, in recent studies, lactate derived from bacteria was shown to mediate small intestinal epithelial proliferation through stimulation of the stem cells in murine organoid cultures (Lee et al., 2018), suggesting there may be specific bacteria-derived factors that interact with the host cells to modulate the ISC response. These findings provide strong evidence for a microbiome role in homeostasis of the ISC niche.

Although the day-to-day regeneration of the luminal epithelium has been well studied and has provided important information, as described above, there is a “catch” to these studies that must be addressed. This regeneration is considered to be homeostatic, meaning that it is an ongoing replacement of the lost cells and whose mechanism is deeply embedded within the physiology of the organ in order to maintain its function. Many researchers differentiate this type of regeneration from the one that takes place following injury to the organ or tissue. Available data support the notion that the mechanisms by which homeostatic regeneration takes place differ from the regeneration that follows injury (Beumer and Clevers, 2016). In this respect, the data shown above relates to the microbiota role in homeostatic regeneration and might not apply to the regeneration of the luminal epithelium under injury or to massive loss due to other manipulations.

In an attempt to understand the ongoing interactions within the digestive tract, invertebrates have been used as simplified organisms. The understanding of the impact of gut microbiota on host physiology has been limited, due to restricted in-depth integrated genetic analysis of both the microbes and the host. In this respect, the study of insect non-binary, yet simpler bacterial communities than mammals, is noteworthy. Intestinal bacterial communities of insects have been widely studied, and the amenability of *Drosophila melanogaster*, allowed its implementation to study animal symbioses. Numerous studies have shown that *Drosophila*’s bacterial communities are simpler than mammals; hundreds of species are present in humans (Qin, et al., 2010), while the adult *Drosophila* midgut symbiotic commensal community is composed of 5–20 different microbial species (Corby-Harris et al., 2007; Cox and Gilmore, 2007; Ren et al., 2007; Ryu et al., 2008; Chandler et al., 2011; Wong et al., 2011). Among them, the families of Acetobacteraceae, Lactobacillales, and Enterobacteriaceae are the most prevalent microbes identified in the *Drosophila* gut microbiota (Ryu et al., 2008; Roh et al., 2008; Chandler, et al., 2011). The simplicity of their microbial communities have made them attractive models for host-microbe studies. Thus, the microbiota effect on intestinal epithelial renewal was studied in *Drosophila*. A crosstalk between the gut and its microbial community was demonstrated to modulate stress response and promote stem cell proliferation and epithelial regeneration (Buchon et al., 2009). Specifically, the pathogenic bacterium *Erwinia carotovora *was shown to be important to undergo intestinal epithelial repairs. This result supports the influence of gut microbiota in epithelial healing, as seen in mammal models. However, unlike vertebrate gut, in *Drosophila*, the intestinal microbiota is composed of either aerotolerant or obligate aerobes, suggesting that oxygen is able to circulate across the *Drosophila* gut (Chandler et al., 2011; Charroux and Royet, 2012). This provides a limitation when comparing the essential compartmentalization that drives the complex ecosystem in humans and non-human vertebrate bodies.

### Leading Studies in Echinoderm Microbial Community

Microbiota has also been associated in echinoderms with other processes important for regeneration such as metabolism and growth. In an early study focused on another echinoderm group, brittle stars, it was suggested that subcuticular and intestinal bacteria could metabolize dissolved organic matter and use it as a significant carbon source (Fielman et al., 1991; Hoskins et al., 2003). These products from microorganisms such as *Pseudoalteromonas atlantica* are proposed to be important for echinoderm physiology, including regeneration processes. The link between microbiota and nutrient availability has also been studied in sea stars, where the need for symbionts’ assistance to ingest structurally complex polysaccharides or require detoxification of dietary products has been suggested (Douglas, 2009). These organic compounds produced by symbionts are potentially used as energy to promote growth and regeneration (Kelly et al., 1995). In another echinoderm species, the purple sea urchin *Strongylocentrotus purpuratus*, studies have also suggested that fasting reduces bacteremia in the coelomic fluid and increases spine regeneration (Scholnick and Winslow, 2020).

*Organ regeneration in echinoderms*- While regeneration of the digestive tract luminal layer has been studied in several model systems, regeneration of the complete intestinal organ has been the focus of work in an understudied group of animals: the Holothuroidea (Echinodermata) (Figure 3B). Several factors make holothurians or sea cucumbers the ideal model system to study the role of the microbiota on regenerative processes. The main one is their ability to eject their digestive tract in a process named evisceration, and to regenerate the entire organ in a period of about a month (Hyman, 1955; Byrne, 2001; Wilkie, 2001; Carnevali, 2006; García-Arrarás et al., 2018). This autotomy, with the subsequent regeneration, provides a unique “natural” model system where the process is part of the animal biology. Moreover, the cellular events that take place during the regeneration of the intestine in these animals have been well studied (García-Arrarás et al., 1998; Quiñones et al., 2002; Mashanov et al., 2005; Candelaria et al., 2006; García-Arrarás et a., 2011; Mashanov and García-Arrarás, 2011; García-Arrarás et al., 2018) and the molecular basis for the regeneration is being actively investigated (Mashanov and García-Arrarás, 2011; García-Arrarás et al., 2018; Quispe-Parra et al., 2021). Echinoderms, being basal deuterostomes, occupy a key branch together with the chordate evolutionary tree, while at the same time are close to most other invertebrates. Moreover, the digestive tube is one of the best conserved organs, common to most metazoans. Thus, these animals can provide useful evolutionary insights into microbiome-host associations. Probably unknown to many, sea cucumbers also have a huge economic value, as part of an aquaculture industry centered in Asia. Thus, the microbiota-host relationships of these animals extend beyond the regenerative process and are studied in terms of health, growth, and other issues related to their nutritional value.

### Comparison of Microbiota Structure Among Sea Cucumber Species

To study the role of the microbiota in intestinal regeneration, we need to first determine the components of the microbiota of our model organisms. Holothurians, as documented in all echinoderms studied to date, have a microbial diversity that is both relatively low and dominated by Proteobacteria. This has been shown in the sub-cuticle of the brittle stars *Ophiactis balli* and *Amphipholis squamate* (Burnett and McKenzie, 1997; Morrow et al., 2018), in the body wall, gonads, pyloric caeca, and coelomic fluid of multiple sea star species (Jackson et al., 2018), in the coelomic fluid, intestines, pharynx, and gut digesta of the sea urchin *Lytechinus variegatus* (Hakim et al., 2015; Hakim et al., 2016; Brothers et al., 2018) and in the intestine of the sea cucumbers *Apostichopus japonicus* and *Holothuria glaberrima* (Gao et al., 2014a; Gao et al., 2014b; Pagán-Jiménez et al., 2019).

**FIGURE 4:**
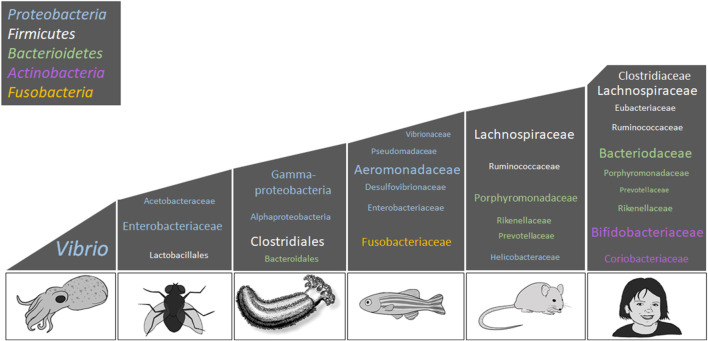
*Bacterial composition associated with animal hosts.* This scheme presents the most representative taxa among the microbiota of *E. scolopes*, *D. melanogaster*, *H. glaberrima*, *D. rerio*, *M. musculus*, and *H. sapiens;* however, relative representation of these taxa may vary per individual. Top phyla among the animal kingdom includes Proteobacteria (blue), Firmicutes (white), Bacteroidetes (green), Actinobacteria (lilac), and Fusobacteria (yellow). The font size represents the relative abundance of the lower taxonomic levels. This figure is an adaptation of Kostic et al. (2013), and contains information from the following studies: Arumugam et al. (2011), Brinkman et al. (2011), Chandler et al. (2011), Roeselers et al. (2011), and Pagán-Jiménez et al. (2019). Images are original drawings by LD-D and AR-V.

The gut commensal microbes of sea cucumbers have been a focus of study during the last decade. The intestinal microbiota of three sea cucumber species: *A. japonicus*, *H. glaberrima,* and *Sclerodactyla briareus* have been described using 16S rRNA gene amplicon sequencing (Gao et al., 2014a; Gao et al., 2014b; Wang et al., 2018; Pagán-Jiménez et al., 2019; Weigel, 2020). Though the Proteobacteria and Bacteroidetes are among the most abundant phyla in all sea cucumber species (Figure 4), a difference in relative representation is seen among different species. Proteobacteria was the predominant phylum within the gut of the holothurian *A. japonicus*, while Gammaproteobacteria was the predominant bacterial class (Gao et al., 2014a; Gao et al., 2014b). A recent study in *S. briareus* supported these findings (Weigel, 2020). In the latter work, the taxonomic representation in the stomach and intestine from animals that were collected from different ponds or aquaria were evaluated and found that the mature intestine microbiota was composed primarily of Proteobacteria. In contrast, our group found that in the intestine of *H. glaberrima,* Firmicutes was the dominant phylum followed by Bacteroidetes, and then Proteobacteria (Pagán-Jiménez et al., 2019). The higher abundance of Firmicutes in *H. glaberrima* may be a key difference with other holothurians, however microbiota differences among holothurians are probably determined by the differences in habitat and/or feeding behaviors (Table 2).

**TABLE 2:** Summary of current findings on sea cucumbers intestinal microbial communities.

Study	*Sea cucumber model*	*Feeding behavior*	*Methods used for library preparation and analysis*	*Samples collected*	*Environmental samples*	*Control groups*	*Intestinal dominant bacteria*	*Regeneration stages*	*Temporal shifts associated to regeneration process*
Gao et al. (2014a)	*A. japonicus* (posterior evisceration)	*deposit feeders*	*16S rRNA gene (V1-V3), 454 sequencing, grouped in OTUs*	*foregut and hindgut contents, and sediment*	*sediment*	*sediment*	*mostly Proteobacteria*	*not applicable*	*not applicable*
Wang et al. (2018)	*A. japonicus*	*deposit feeders*	*16S rRNA gene (V3-V4), Illumina HiSeq, grouped in OTUs*	*foregut, midgut, and hindgut with cloaca*	*none*	*1*) *non-eviscerated animals at the initial stage of experiment (plus 4days in “template culture”) and 2*) *non-eviscerated animals at the final stage (55days)*	*Proteobacteria, Bacteroidetes, Euryarchaeota, and Firmicutes*	*1-, 5-, 15-, 25-, 35-, 45-, 55- dpe*	*Earlier regeneration stage (1–25- dpe): Proteobacteria in all samples, yet the sub-dominant phyla were different between samples. Later regeneration stage: (35–55- dpe) Proteobacteria and Bacteroidetes and the relative abundance of both reached above 95%*
Zhang et al. (2020)	*A. japonicus*	*deposit feeders*	*16S rRNA gene (V4–V5), Illumina HiSeq, grouped in OTUs*	*whole intestines of regenerating animals*	*not applicable*	*intestines of non-eviscerated sea cucumbers*	*Proteobacteria, Bacteroidetes, and Firmicutes*	*10-, 14-, 18-, and 21- dpe*	*Bacteroidetes’ relative abundance increased on day 14 and day 18*
Yamazaki et al. (2016)	*A. japonicus*	*deposit feeders*	*16S rRNA gene (V1–V2) grouped in OTUs and ASVs*	*feces of eviscerated animals at different time points*	*not applicable*	*1)* *feces of non-eviscerated sea cucumbers at different time points and 2*) *feces from all animals at time point 0 (pre-evisceration)*	*families were explored: in most samples Rhodobacteraceae is dominant followed by Alteromonadaceae*	*samples collected at different time points, mainly 15-, 16-, 17-, 20-, 24-, and 28- dpe*	*The pre-evisceration fecal microbiota is significantly different from that of the feces post-eviscerationOne animal had a high abundance of the family* Colwelliaceae *in the feces collected pre-evisceration, yet the abundance drastically decreased after gut regeneration (around 17- dpe). Same thing happened with* Flavobacteriaceae *and* Rhodobacteraceae *with other two samples, respectively*
Pagán-Jiménez et al. (2019)	*H. glaberrima* (posterior evisceration)	*suspension feeders*	*16S rRNA gene (V4-V5), 454 sequencing, grouped in OTUs*	*anterior, medial, posterior, and seawater*	*seawater*	*1*) *seawater and 2*) *tissues from* *animals dissected in situ*	*Proteobacteria, Bacteroidetes, Fusobacteria, and Firmicutes*	*not applicable*	*not applicable*
*Weigel (2020)*	*S. briareus* (anterior evisceration)	*deposit feeders*	*16S rRNA gene (V4), Illumina HiSeq, grouped in amplicon sequence variants (ASVs)*	*stomach and medial small intestine for mature and control individuals, stomach and whole intestine for regenerating individuals*	*seawater, sediment, algae Gracilaria sp*., and *seagrass Zostera marina*	*1*) *initial condition control: samples of stomach and intestines after 2-days lab acclimation*	*mostly Proteobacteria in mature intestines. Mature intestines from tank control also had a high percentage of Epsilonbacteraeota*	*13-, 17-, 20- dpe*	*The regenerating intestine microbiomes were dominated by Proteobacteria, Bacteroidetes, and Epsilonbacteraeota similar to mature intestines (initial and tank controls) but have a higher diversity in lower taxonomic levels. Richness increased from evisceration to day 20dpe. regenerating intestines had higher evenness than mature intestines*
						*2*) *tank control: stomach and intestine of animals eviscerated after 18 days in the tank without evisceration*			

dpe, day post evisceration.

Apart from differences among the gut microbiota of various species, discrepancies in the microbiota between areas of the gut have been observed. Some of these differences are seen in the relative abundances of the microbial community in various segments of the digestive tract. In *H. glaberrima* dissected *in situ* (as soon as collected from the intertidal space), both areas of the small intestine (comprised of anterior and the medial) showed a similar microbiota, composed mostly by members of the phylum Proteobacteria (Pagán-Jiménez et al., 2019). However, the large (posterior) intestine contained mostly Firmicutes. Beta analysis supported these results, revealing that the anterior, medial, and posterior intestine samples had significantly different microbial communities. In addition, differences between environmental microbiota and gut microbiota were documented. Both seawater microbial communities (collected *in situ* and the aquarium water) were more similar to the communities of the anterior and medial intestine, than that of the posterior intestine. These data suggested a distinctive microbiota in the large intestine. In species where the digestive tract includes a stomach, different bacterial communities were found between the stomach and the intestine (Weigel, 2020). Similarly, different microbiotas were found between the water and the internal organs. Thus, all published studies of sea cucumbers microbiota show significant differences between the seawater and the intestinal communities, and differences among the digestive tract structures themselves (Gao et al., 2014a; Gao et al., 2014b; León-Palmero et al., 2018; Pagan-Jimenez et al., 2019; Weigel, 2020). This contrast between marine animal organs and seawater microbial communities was found in other organisms including corals, sea urchins, sea stars and sea anemones (Ainsworth et al., 2015; Brothers et al., 2018; Jackson et al., 2018; León-Palmero et al., 2018).

It is imperative to mention that the experimental design for studies of the microbial communities in holothurians, including the dissections and tissue collections varied among the different studies. In some, the viscera were processed individually, while in others the intestine was not separated from the cloaca. Thus, in Table 2 we summarized the similarities and contrast of holothurian microbiota studies.

### Examining the Microbe-Echinoderm Associations

In *A. japonicus*, the link between microbial diversity and animal growth has been examined (Yamazaki et al., 2016). The metagenomes of feces of large and small sea cucumbers were sequenced, showing that larger and smaller animals had different microbiota, and that while the alpha diversity was similar, the relative abundance differed. The orders Rhodobacterales, Oceanospirillales, and Desulfobacterales were more abundant in larger animals. The long-term effects, in terms of growth and disease resistance, of disrupting the bacterial community of *A. japonicus* sea cucumbers by using antibiotics was also explored (Zhao et al., 2019). Interestingly, after administering different antibiotics (tetracycline, erythromycin, or norfloxacin), it was observed that some antibiotics increased the growth of the animals yet weakened their immunological system. In a different study, Yamazaki and colleagues found that *Rhodobacterales* are the third most abundant order in the fecal microbiota of *A. japonicus,* and the relative abundances were significantly higher in larger animals than in smaller individuals (Yamazaki, et al., 2016). However, the subsequent article by Zhao and colleagues reported a decreased relative abundance of Rhodobacteraceae in *A. japonicus* juveniles when treated with either tetracycline or erythromycin, but an increase in sea cucumber survival and body weight (Zhao et al., 2019). The above summarized studies demonstrate how bacteria metabolic activity might play a key role in providing the energy source to hosts to facilitate or activate their cellular and molecular process.

### Is the Echinoderm’s Regenerative Capacity Influenced by the Microbiota?

Two types of studies explore if the microbiome influences the intestinal regeneration of holothurians. The first group of studies focuses on correlating changes in the microbiome with different stages of intestinal regeneration. The regenerating gut microbiome of *A. japonicus* was characterized, showing that Proteobacteria (Wang et al., 2018) or Actinobacteria (Zhang et al., 2020) were the dominant phyla from wound healing stage to lumen formation (early in their regeneration process), and at later stages of regeneration, Proteobacteria and Bacteroidetes became the dominant phyla (Wang et al., 2018; Zhang et al., 2020). This change suggested that during early stages of the regeneration process*,* the gut was populated mostly by the bacteria from the sediments and water, and then was gradually replaced by digestive-associated microbiota.

An expanded analysis of regenerating intestine microbiota in a different holothurian species, *S. briareus,* documented higher richness (on day 20 after evisceration) and evenness (on day 13–20 after evisceration), when compared to mature intestines (Weigel 2020). Moreover, an Alphaproteobacteria species abundant in mature intestine samples was not found in regenerating intestines. Regenerating stomachs were found to be more diverse in comparison to mature ones. Interestingly, beta-analysis plots showed that regenerating stomach and regenerating intestine were similar. Taxonomic representation and alpha diversity analysis revealed that the regeneration process was associated with a change in microbial community that recovered at the end of the regeneration process. In addition, tank residence, but not collection site, were suggested to affect gut microbial community, however changes in the regenerating microbes were not simply due to tank effects.

It is important to highlight these studies because they propose a correlation between the gut microbiota and the regeneration process. However, as mentioned before, these findings were shown mainly by using functional inferences from genomic data which do not strongly establish that the microbial community causes a particular effect ruling the intestinal regeneration associated events. For example, genomic data cannot distinguish if the organisms found in this community were even alive or if they were transient (ingested debris or indiscriminate colonization). Thus, the future of this field is beyond correlative analysis, and it requires experimentation that delves directly into the microbial community influence and if its modulation alters the effects on host’s regeneration.

The second type of study, which precisely examined the role of the microbiome in holothurian gut regeneration, was recently published by our group (Díaz-Díaz, et al., 2021). Here, different antibiotic cocktails were used to cause dysbiosis and study the influence of the commensal community in the intestinal regeneration process. We observed that antibiotic treatments altered cellular processes associated with regeneration such as cellular dedifferentiation, extracellular matrix remodeling, and cell proliferation. To rule out that the antibiotics were exerting a direct effect on the holothurian tissues, we performed MTT assays on dissociated cells and explant cultures. *Ex vivo* experiments suggested that the antibiotics used did not directly alter the holothurian tissue metabolic activity, while being capable of inhibiting gut bacterial populations *in vitro.* Therefore, we proposed that the antibiotics are influencing *H. glaberrima* regeneration via the dysbiosis of the gut microbiota. Moreover, because *H. glaberrima* microbiota is mainly composed of Firmicutes (mostly Gram-positive bacteria) and Proteobacteria (mostly Gram-negative bacteria) (Pagán-Jiménez et al., 2019), and the cocktails targeting mostly the Gram-positive bacteria had the most detrimental effects over the intestinal regeneration, we suggest that Firmicutes may have a crucial role in the progression of the intestinal regeneration. Antibiotics have also been shown to have long-term effects on holothurian growth and disease resistance. In an experiment where antibiotics (tetracycline, erythromycin, or norfloxacin) were administered to disrupt the bacterial community, some antibiotics increased the growth of sea cucumbers, yet appear to inhibit the animal immune system (Zhao et al., 2019).

Furthermore, the role of the microbiota during regeneration could be addressed using other echinoderms. The crinoids, which are well known for their potential to regenerate their arms, can also lose and renew their entire digestive system (Dendy, 1886; Meyer, 1985; Meyer, 1988; Mozzi et al., 2006; Bobrovskaya and Dolmatov, 2014; Kalacheva et al., 2017). These studies describe the fast visceral regeneration potential in crinoids, such as *Antedon mediterranea*, *Antedon rosaceus*, *Himerometra robustipinna* and *Lamprometra palmata*, through histological and cytological analysis but were neglected for many years. We propose that echinoderms are promising models to elucidate if, and how, the regeneration events in the digestive system are influenced by the gut microbiota. Moreover, these organisms provide models whose findings on the whole organ regrowth are not limited solely to the study of the repair of the luminal epithelium layer of the intestine.

Nevertheless, we acknowledge that these models have some disadvantages. First, one deficiency for many of the echinoderms members is the lack of genomic and metagenomic data available. Second, as they are marine invertebrates, the structure and function of their microbiota might be very distinctive, in comparison to humans; colonized by species that are not observed in terrestrial vertebrates.

## Conclusion

Microbiota effects on regenerating tissues are just beginning to be investigated. The initial findings strongly suggest that, indeed, bacterial species composition is an important factor in the timing and effectiveness of the regenerative process. However, most of the available data is correlative and needs to be backed by functional studies. These correlative studies on microbial successions and the regeneration process do not demonstrate a causal effect on the intestinal regeneration exerted by the gut microbiota. Nonetheless they do provide some evidence that supports the hypothesis that the microbiota may be influencing regenerative events.

The challenge for future investigations is to identify the specific roles of the microbiota and the signaling pathways or physiological processes by which they might modulate regeneration. Central to this issue is the use of appropriate model systems in which to decipher the specifics of the microbe associations. We consider that *in vivo* examinations where the use of agents that modulate the microbiota, such as prebiotic, probiotic or antibiotics, will be crucial to understand the role of these microorganisms during gut repair mechanisms. Here, we have described various promising echinoderm models to decipher the role of the microbiota during intestinal regeneration, that encompasses the whole organ formation beyond the luminal epithelium repair and homeostasis. We propose that this need may be fulfilled in part by the sea cucumber intestinal regeneration model. The fact that the regenerating organ is a structure present in most metazoans and is the one organ where most microbiome studies have been made, makes this model particularly attractive to study host-microbiome interactions. Thus, we expect that studies with holothurians will provide groundbreaking knowledge on the field of microbiome-host associations and their impact on regenerative processes. However, this model also has some limitations. Among them, the need to improve the molecular tools available to study the specific functions of certain genes as well as the present limitations on identifying and characterizing many bacteria (and other components of the microbiota) that are difficult or impossible to grow in the laboratory. Nonetheless, we believe that comparative studies using the sea cucumber, as well as other models, will be transformative in defining the interactions of host-microbiome in regenerative processes.
